# Selected Biomarkers Revealed Potential Skin Toxicity Caused by Certain Copper Compounds

**DOI:** 10.1038/srep37664

**Published:** 2016-11-28

**Authors:** Hairui Li, Pei Zhen Toh, Jia Yao Tan, Melvin T. Zin, Chi-Ying Lee, Bo Li, Melvina Leolukman, Hongqian Bao, Lifeng Kang

**Affiliations:** 1 Department of Pharmacy, National University of Singapore, 18 Science Drive 4, 117543, Singapore; 2 3M Innovation Singapore, 100 Woodlands Avenue, 738205, Singapore

## Abstract

Copper is an essential mineral and plays important roles in skin growth and activity. Copper delivery through skin can provide beneficial effects but its potential to induce skin irritation reactions is often overlooked. Data on dermal toxicity caused by copper compounds is scant. Some recognized *in vitro* skin toxicity methods are unsuitable for all metal compounds. Here, we employ a keratinocyte-based model and evaluated the skin irritation potential of copper compounds at cellular, genomic and proteomic levels. We determined cell viability and cytotoxicity by using tetrazolium reduction assay and Lactate Dehydrogenase (LDH) assay, performed real-time PCR and protein quantification to assess the expression of biomarkers after treating cells with copper peptide (GHK-Cu), copper chloride (CuCl_2_) and copper acetate (Cu(OAc)_2_). These copper compounds exhibited different irritancy potentials at the same treatment concentrations. GHK-Cu was not cytotoxic and did not induce any significant change in the expression levels of various skin irritation-related biomarkers. IL-1α and IL-8, HSPA1A and FOSL1 were significantly upregulated following 24-h treatment with CuCl_2_ and Cu(OAc)_2_ at 58 and 580 μM without concomitant inhibition in cell viability. GHK-Cu has a low potential of inducing skin irritation and therefore provides a safer alternative for the delivery of copper through skin.

Copper is an essential trace element critical for normal human metabolism. Copper deficiency can occur with inadequate copper intake, but excess copper intake may cause toxicity to human. Copper and its alloys are present in numerous articles of everyday use, such as coinage, tools, jewelry, and dental materials, thus it comes in regular, sometimes extended contact with skin. For medical applications, copper intrauterine devices are a type of long-acting contraceptive because copper (II) ions released from the devices are deleterious to sperm cells^1^. Direct and prolonged contact of copper with skin may result in electrochemical reactions to release copper ions, which are diffusible through the skin^2^. Copper in its metallic state has no effect on the skin and it becomes a potential irritant or allergen when it is corroded to become soluble through the action of exudates encountered on the skin surface, or in a relatively corrosive physiological environment such as the oral cavity or the uterus^3^.

Historically, copper compounds have been used as paint pigments, wood preservatives and pesticides^4^. Copper compounds are also used as actives or excipients in cosmetic and pharmaceutical products, e.g., copper peptide for skin regeneration purposes, cupric aspirinate and cupric salicylate for rheumatoid arthritis treatment, copper conjugated dendrimer for anti-tumoral activity, and copper liposome for enhanced stability of doxorubicin^5^,^6^,^7^,^8^,^9^. Copper can also be used in photo-thermal nanoparticles. These copper nanoparticles-mediated drug delivery systems can significantly increase the permeability of drug and enable controlled transdermal drug delivery^10^. With developments in novel skin permeation enhancing methods such as microneedles and laser, copper compounds can potentially be used on skin for cosmeceutical or medicinal purposes^5^,^11^,^12^. Efficacy and safety studies are equally important for the successful development of such delivery systems. However, many current studies are primarily focused on improving the pharmacodynamic and pharmacokinetic properties of copper, while data on copper-induced dermal toxicity remain limited. Along with wide exposure of copper on the skin or mucosa, incidences of contact dermatitis have been reported although not frequently. For instance, a woman who reported a 5-year history of painful lichenoid lesions on the left mucosa and on the left side of the tongue adjacent to a dental metal prosthesis containing copper, had all these symptoms relieved after the prosthesis was substituted to one without copper. Dermatitis and eczematous rash were also reported by individuals who used IUD. Hand eczemas were commonly reported among cashiers and other professionals handling coins, which may be caused or aggravated by the release of metal ions including copper^3^. In essence, sources of copper-induced irritations can range from occupational exposures to copper-based items to copper-based pesticides, resulting in allergic reactions, such as itching and eczema^13^.

Considering the wide exposure to copper and its compounds in our daily life, more information regarding copper compounds on skin toxicity becomes relevant. However, data on dermal irritation by copper and its compounds are scant and the role of copper as an irritant/sensitizer remains controversial^3^. A recent review on copper hypersensitivity concluded that copper is a weak sensitizer as compared with other metallic compounds^14^. However, with the prevalence of skin permeation enhancement methods (such as microneedles and laser) used in cosmetic and pharmaceutical industry^15^, copper compounds may pose a higher risk to cause skin irritation, which necessitates a thorough skin toxicity testing. Animals tests for acute skin irritation assessment usually follow the Draize rabbit test while other accepted assays for skin sensitization include Local Lymph Node Assay (LLNA) and Guinea Pig Maximisation Test (GPMT). However, LLNA is deficient in detecting metals and organometallic compounds^16^. Furthermore, due to questionable significance of animal data and ethical opposition to animal testing, effort has been to put into finding alternative testing methods to identify potential skin toxins^17^,^18^,^19^. Most considerations of non-animal alternatives for skin irritation/sensitization testing tend to examine a single aspect of the process (e.g., chemical reactivity, epidermal bioavailability, dendritic cell responses). However, experts in the area generally concur that a combination of data from multiple endpoints is needed to discriminate sensitizing and/or irritant substances *in vitro*. Besides, Direct Peptide Reactivity Assay (DPRA) is the assay recommended by EURL ECVAM for skin sensitization testing. However, DPRA is not suitable for metallic compounds, because they may form bonds with the nucleophilic residues in histidine^20^.

Hence, we are interested to identify multiple biomarkers to evaluate the skin irritancy potential of various metallic compounds, specifically, copper compounds. Keratinocytes make up 95% of the cells in the epidermis and play an integral role in regulating skin irritation. It was found that human keratinocyte cell line closely resembles normal keratinocytes in their growth and differentiation characteristics, hence allowing accurate prediction of response after treatment^21^,^22^. After exposure to an irritant, the irritant will exert toxic effects on keratinocytes. This will activate the body’s innate immunity with the release of cytokines such as interleukin 1, alpha (IL-1α) in the keratinocytes. In turn, these cytokines will activate Langerhans cells, dermal dendritic cells and endothelial cells, which contribute to cellular recruitment to the site of insult. Neutrophils, lymphocytes and macrophages will then be released to further promote the inflammatory cascade^23^. These will lead to various symptoms of irritation such as itch, erythema, edema and pain. By monitoring the inflammatory responses, such as cytokine secretion and receptor activation, various biomarkers can give us an indication of the possibility of a safe and efficient transport of copper through skin to exert its beneficial effects.

Currently, endpoint measurements are related to severe cell damage such as tetrazolium reduction assay and lactate dehydrogenase (LDH) assay to detect viable cells and IL-1α release as inflammatory markers^21^. Important early changes occur before severe cell damage take place but no universal markers have yet been identified^21^. The search for new endpoints is necessary, given the complexity of skin irritation mechanism. Various potential biomarkers have been studied and identified using proteomic and toxicogenomic technologies^24^,^25^,^26^. These technologies potentially allow the setup of an *in vitro* test system, which resembles the *in vivo* situation as closely as possible^27^.

In this study we investigate the safety of copper complex of glycyl-L-histidyl-L-lysine (GHK-Cu), copper chloride (CuCl_2_) and copper acetate (Cu(OAc)_2_) by using *in vitro* skin irritation tests. Based on the mechanism of skin irritation, we examined and proposed an approach to assess the skin irritation potential of copper compounds using cytotoxicity assay, gene and protein expression levels of cytokines. To our knowledge, this is the first study evaluating the irritancy potential of different types of copper compounds at the cellular, genomic and proteomic levels using keratinocyte cell lines. This approach may also be useful to assess the irritancy potential of other inorganic compounds or minerals in the cosmetological, dermatological and pharmaceutical applications.

## Results

### Cell Viability Assay

**Figure 1:**
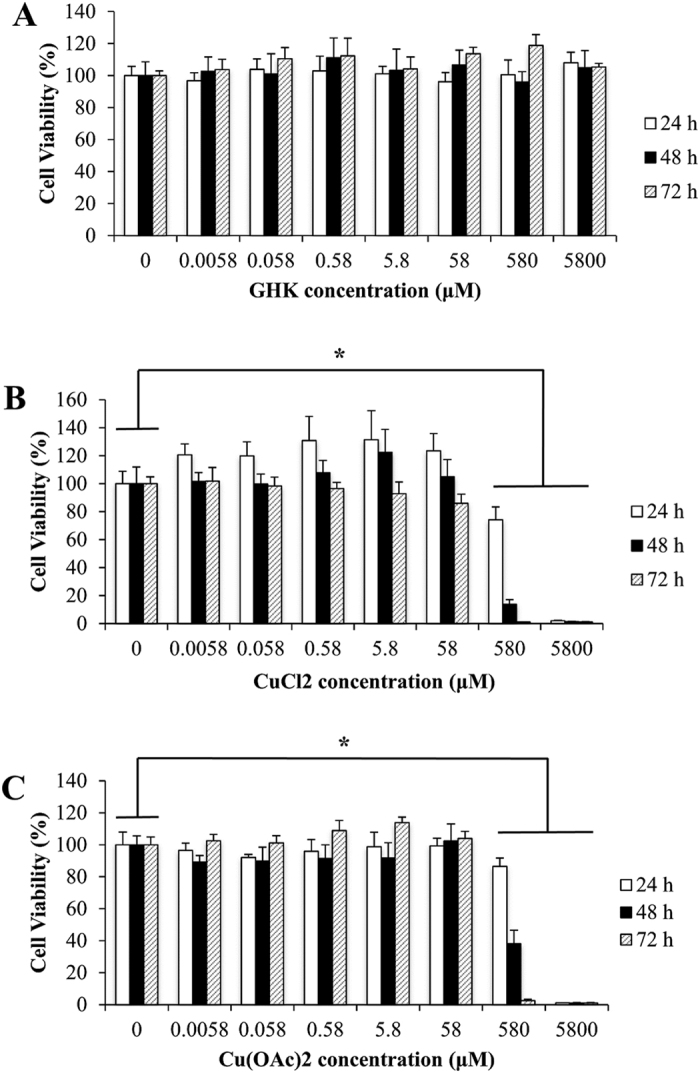
Cell viability assay. Relative cell viability of HaCaT keratinocytes after incubation with GHK (**A**), CuCl_2_ (**B**), Cu(OAc)_2_ (**C**) for 24, 48 and 72 hours. ANOVA was performed between the control and treatment groups followed by Tukey’s post hoc test. *p < 0.05, is considered to be statistically significant compared with control.

Cell viability tests were performed on HaCaT keratinocytes cells with the treatment of GHK, CuCl_2_ and Cu(OAc)_2_ for 24, 48 and 72 h. Cell viability for GHK-Cu was previously investigated by Hairui et al, showing no significance difference between the control and the GHK-Cu treated group at the same concentrations and time points, implying the non-cytotoxic nature of GHK-Cu towards HaCaT^11^. After treatment with concentrations ranging from 0.0058 μM to 5800 μM, cell toxicity was not observed for GHK treated group (Fig. 1A). On the other hand, the toxic effects were observed for groups treated with CuCl_2_ and Cu(OAc)_2_. The cytotoxic effects of these copper compounds on HaCaT cells were observed to be concentration-dependent and time-dependent at concentrations 580 μM and 5800 μM. Time-dependent cytotoxicity was most evident at 580 μM as the cell viability was greatest at 24 h, followed by 48 h and the least at 72 h. Concentration-dependent cytotoxicity was observed as 5800 μM gave a lower cell viability as compared to 580 μM. At the highest concentration of 5800 μM, both CuCl_2_ and Cu(OAc)_2_ were found to be very cytotoxic to the cells and almost all the cells were dead at the three time points. The reduction in overall cell viability was statistically significant for both CuCl_2_ (Fig. 1B) and Cu(OAc)_2_ (Fig. 1C) treated groups at 580 μM and 5800 μM.

### Lactate Dehydrogenase (LDH) Assay

**Figure 2:**
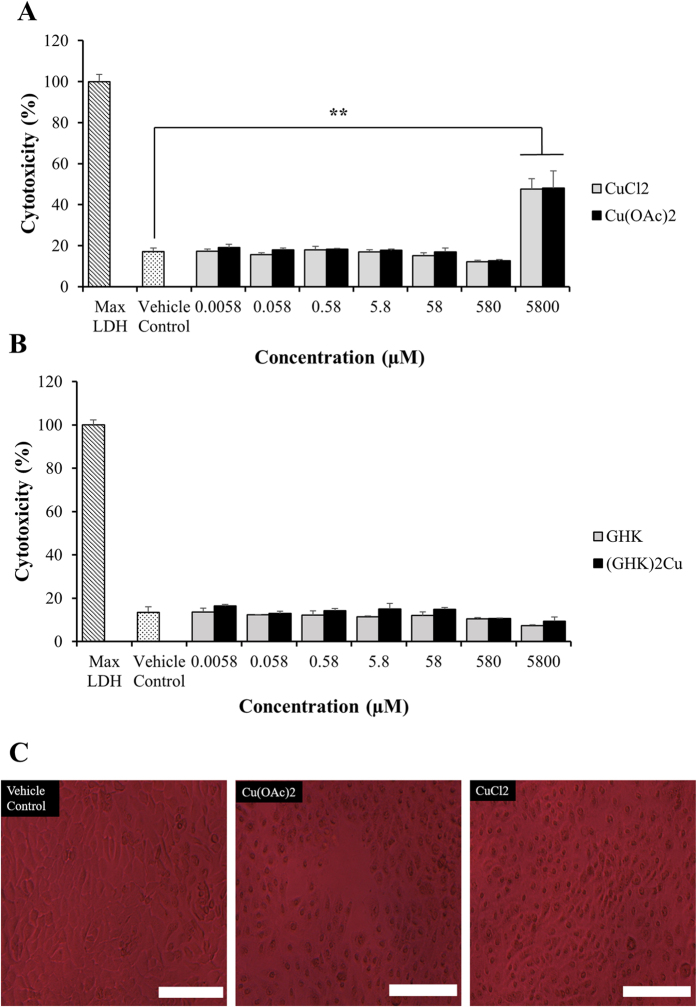
Lactate Dehydrogenase assay. Percent (%) cytotoxicity of Cu(OAc)_2_ and CuCl_2_ (**A**), GHK and GHK-Cu (**B**) on HaCaT keratinocytes after 30 min treatment followed by 24 h incubation with serum-free media. Student’s t-test was performed between the control and treatment groups. *p < 0.05 versus control; **p < 0.01 versus control. Signs of necrosis (cells shrinking and rounding) observed in HaCaT keratinocytes treated with Cu(OAc)_2_ and CuCl_2_ after 30 mins (**C**). (Scale bar = 200 μm).

In this assay performed on HaCaT keratinocytes cells, the cells were treated with GHK, GHK-Cu, CuCl_2_ and Cu(OAc)_2_ for 30 mins at 7 different concentrations each, ranging from 0.0058 to 5800 μM–0.0058 μM, 0.058 μM, 0.58 μM, 5.8 μM, 58 μM, 580 μM, 5800 μM. CuCl_2_ and Cu(OAc)_2_ were significantly more cytotoxic (*p* < 0.05) to the HaCaT keratinocytes at 5800 μM (Fig. 2A) as compared to GHK, GHK-Cu (Fig. 2B). At concentrations below 5800 μM, no sign of cytotoxicity was observed in cells treated with all the 4 copper compounds. Characteristics of cellular necrosis – cell rounding and shrinking - in the HaCaT keratinocytes were observed in samples treated with 5800 μM of CuCl_2_ and Cu(OAc)_2_ after 30 min. (Fig. 2C).

### Quantitative Real-Time PCR Analysis

Real-time PCR was then performed to determine the expression of 8 genes, which were involved in skin irritation and inflammation. These genes were previously found to be upregulated in the presence of irritants^24^,^28^. The fold changes in relative gene expression of treatment cells compared to sterile water treated control cultures were determined following 24 h of treatment.

**Figure 3:**
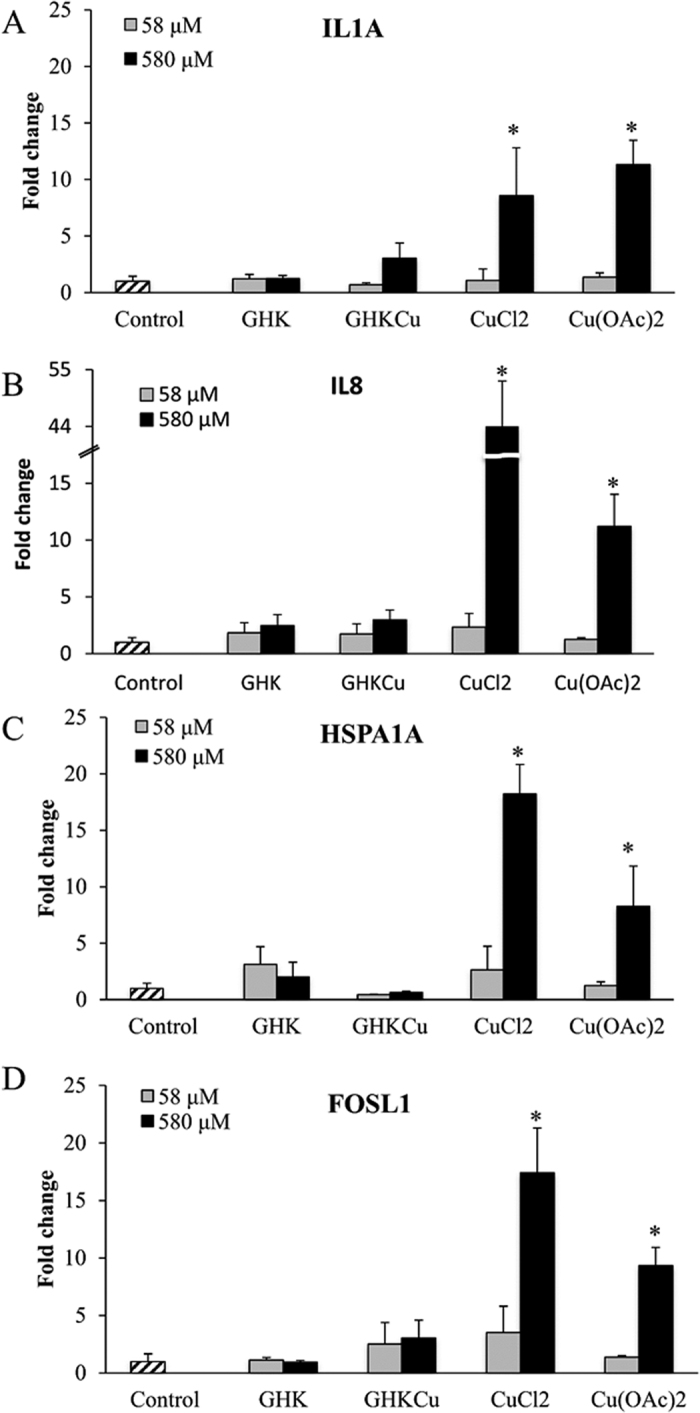
Real-time PCR analysis on expression of IL1α (**A**), IL8 (**B**), HSPA1A (**C**), FOSL1 (**D**) following treatment for 24 h in HaCaT keratinocytes. The fold change was calculated as the normalized ratio in treatment cells compared to that in control. ANOVA was performed between the control and treatment groups for each gene followed by Tukey’s post hoc test. *p < 0.05, is considered to be statistically significant compared with control.

From SI 1, it was shown that heat shock protein 27 (HSP27), superoxide dismutase 1 (SOD1), cofilin 1 (CFL1) and bone morphogenetic protein 2 (BMP2), did not show any significant change in expression levels among all the treatment groups with *p* > 0.05. While the change in gene expression of the other four genes, interleukin 1 alpha (IL1A), interleukin 8 (IL8), FOS-like antigen 1 (FOSL1) and heat shock protein 70 kDa 1A (HSPA1A) were significantly different among the control and treatment groups (*p* < 0.05), which were further plotted in Fig. 3.

From Fig. 3, GHK and GHK-Cu at 58 μM and 580 μM did not induce any significant change in the expression of all the tested genes compared to the control (*p* < 0.05).

For CuCl_2_ and Cu(OAc)_2_, the gene expression levels were concentration-dependent, while the treatment concentration of 580 μM induced a higher expression of these genes than that of 58 μM. At 58 μM, CuCl_2_ and Cu(OAc)_2_ didn’t induce any significant upregulation of the four genes. After treating HaCaT with 580 μM CuCl_2_ or Cu(OAc)_2_, the expression of IL1A, IL8, HSPA1A and FOSL1 were significantly higher than that in the control group and ranged from a 8–44 fold change. Specifically, 580 μM CuCl_2_ induced the upregulation of IL8 up to 44 times. At the same concentration of 580 μM, Cu(OAc)_2_ induced a higher upregulation of IL1A than CuCl_2_, while CuCl_2_ induced a higher upregulation of IL8, HSPA1A and FOSL1 than Cu(OAc)_2_.

### Measurement of Protein Expression by ELISA

**Figure 4:**
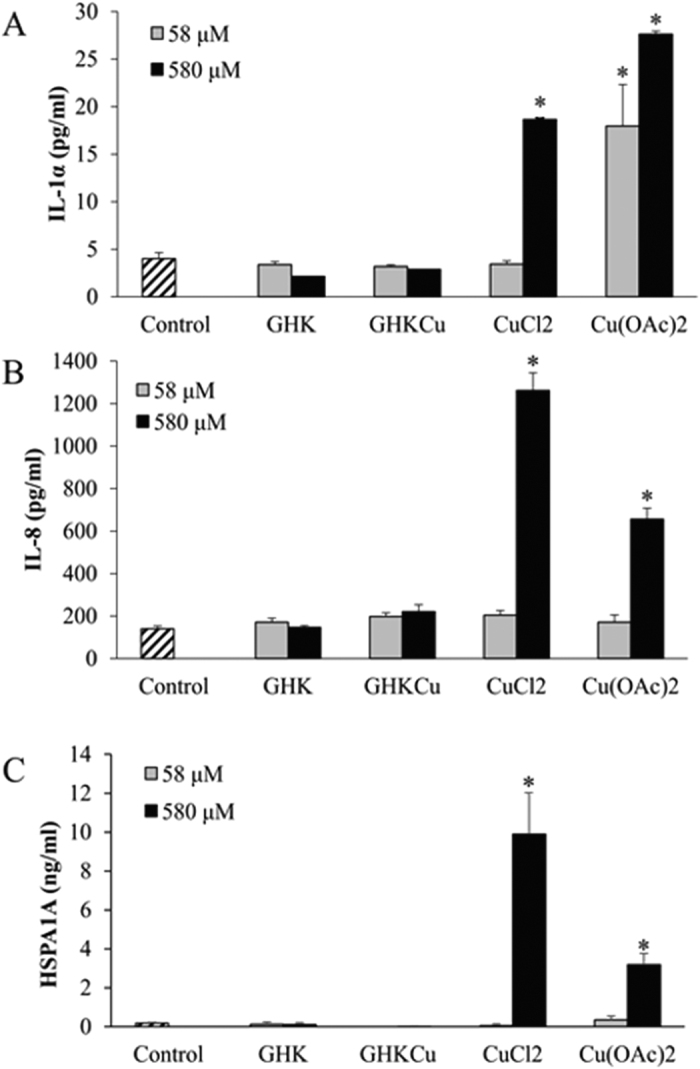
Concentration of IL-1α (**A**), IL-8 (**B**) and HSPA1A (**C**) in cell culture medium quantified by ELISA. Results were presented as pictogram or nanogram of mediator released per milliliter of conditioned medium. ANOVA was performed between the control and treatment groups followed by Tukey’s post hoc test. *p < 0.05, is considered to be statistically significant compared with control.

The extracellular content and release of IL-1α, IL-8, HSPA1A and FOSL1 were determined using ELISA. The expression of FOSL1 in the culture medium for all the samples were undetectable. The concentrations of IL-1α, IL-8 and HSPA1A released after treatment with various compounds were presented in Fig. 4.

At the two tested concentrations, treatment with both GHK and GHK-Cu did not significantly increase the IL-1α, IL-8 and HSPA1A protein levels. For the treatment groups with CuCl_2_ and Cu(OAc)_2_, copper compounds had a positive correlation with the protein level regulated.

CuCl_2_ didn’t cause any change in the IL-1α, IL-8 and HSPA1A protein levels at the concentration of 58 μM, while the protein levels upregulated significantly with 580 μM CuCl_2_ treatment. On the other hand, the IL-1α, IL-8 and HSPA1A protein levels were increased significantly with 580 μM Cu(OAc)_2_ treatment as well. At the same concentration of 580 μM, Cu(OAc)_2_ induced a higher upregulation of IL-1α than CuCl_2_, while CuCl_2_ induced a higher upregulation of IL-8, HSPA1A and FOSL1 than Cu(OAc)_2_. Furthermore, 58 μM Cu(OAc)_2_ also upregulated the IL-1α expression significantly.

## Discussion

Copper is an essential mineral and has many potential benefits in skin growth and activity^29^. Copper delivery through the skin has many potential applications and thus is widely studied, including the evaluation of the penetration ability of copper peptides^30^, biocompatibility of copper containing IUD^1^, the enhancement of skin absorption through copper-containing nanoparticles^10^ and microneedle-mediated delivery^5^. Hence, the safety associated with copper delivery through the skin needs to be established. In this study, we investigated the skin toxicity of several copper compounds using cell-based *in vitro* tests and evaluated the expression of various cytokines at genomic and proteomic levels to discover irritancy biomarkers suitable for copper compounds.

Before the cell testing, we analyzed the pH of the samples tested and we excluded the pH of copper compounds as the influencing factor of their potential cytotoxicity (SI 2). Subsequently, HaCaT keratinocytes’ viability after treatment with test compounds, GHK, CuCl_2_ and Cu(OAc)_2_ were assessed using MTT assay and LDH assay. Cultured human keratinocytes have been suggested as good models to predict dermal irritancy of substances in human subjects^31^. Primary keratinocyte culture is limited by their variability in culture conditions, and their susceptibility to irritants varies with the number of passages. On the other hand, the non-tumorigenic, spontaneously immortalized keratinocyte cell line - HaCaT cells can provide almost unlimited supply of identical cells, ensuring high intra- and inter-laboratory reproducibility. Besides, *in vitro* cytotoxicity with HaCaT keratinocytes and human *in vivo* data was well- correlated^22^,^32^,^33^. HaCaT keratinocytes have also been proven to be good candidates for assessing skin irritancy and toxicity in conventional monolayer assays measuring using MTT reduction as the viability endpoint^21^.

MTT assay mainly reflects the damage to cellular mitochondria, and it is commonly used for *in vitro* cytotoxicity (including skin irritation) measurement. Our results indicated that neither GHK nor GHK-Cu showed cytotoxic activity after 72 h exposure to HaCaT cells. CuCl_2_ and Cu(OAc)_2_ were only found to be cytotoxic at 5800 μM and cell viability decreased significantly after 48 h treatment at 580 μM. At 58 μM and 580 μM, cytotoxicity was not observed following 24 h treatment with all the samples. The results of the MTT assay were consistent with the results of LDH assay as CuCl_2_ and Cu(OAc)_2_ were able to induce significant cytotoxicity in the HaCaT keratinocytes within 24 hours, but not for GHK and GHK-Cu. Lactate dehydrogenase (LDH) is a cytosolic enzyme and its presence in the media is often used as an indicator for cellular toxicity. Large amount of LDH in the media is linked to the leaky, damaged and compromised cellular membrane integrity. The measurement of LDH in media has therefore been a commonly used and validated method for assessing cytotoxicity among many *in vitro* based cell model studies. The LDH assay was performed to validate the results obtained from the MTT assay. In this assay, the 30 min incubation duration was chosen to be optimal because of the inherently cytotoxic nature of CuCl_2_ and Cu(OAc)_2_ towards HaCaT keratinocytes, as they were observed to cause substantial cell death at a duration longer than 30 mins. This may result in a false negative result as the media containing the LDH was replaced with fresh serum-free media and incubated for another 24 h and most of the LDH released into the media would have been removed in the process. The replacement of the media is essential for removing the CuCl_2_ and Cu(OAc)_2_ interference before assaying the LDH levels as they are shown to reduce the fluorescence signal read-out within treated wells. Our results indicated that neither GHK nor GHK-Cu showed cytotoxic activity at the range of concentrations tested while CuCl_2_ and Cu(OAc)_2_ showed substantial cytotoxicity to the HaCaT cells at the highest concentration assayed, i.e., at 5800 μM, after 30 mins treatment.

Another study with CuCl_2_ treated Hep G2 cell line demonstrated that mitochondrial toxicity is an intermediate-level event associated with copper toxicity to Hep G2 cells while lysosomal damage is the early event and plasma damage a late feature associated with copper toxicity^34^. Considering that the copper-associated mitochondrial toxicity may have been delayed, we further explored the changes in the expression of skin irritation-related biomarkers at the genomic level. In response to chemical stress, keratinocytes produce and release inflammatory cytokines, chemokines and other signaling markers that rapidly generate cutaneous inflammation^35^. Among the genes evaluated, expression levels of genes were not significantly different in GHK and GHK-Cu group as compared to control. However, we found that four genes, IL-1α, IL8, HSPA1A and FOSL1, were significantly induced by CuCl_2_ and Cu(OAc)_2_ without inhibiting the cell viability. The protein expression of the four genes in culture medium were further tested with ELISA kits. With the exception of FOSL1, which was undetectable in all of the samples in the ELISA test, the protein level regulation of IL-1α, IL8, HSPA1A by the copper compounds were similar with the gene level regulation. GHK and GHK-Cu didn’t cause any significant changes in the extracellular protein concentration of IL-1α, IL8, HSPA1A. However, CuCl_2_ and Cu(OAc)_2_ significantly upregulated the extracellular protein expression of the three genes at concentrations (58 μM and 580 μM) without inhibiting the cell viability after 24 h.

Cytokine production and response are important as they participate in immune and inflammatory responses. Specifically, IL-1α is constitutively produced and retained in keratinocytes, but in response to several stimuli, IL-1α is released, which is essentially a primary event of inflammation. Triggers of IL-1α production have been observed in a number of different stimuli, such as sulfur mustard, nickel, contact allergens or superantigens^36^ IL-1α stimulates further release of secondary mediators, including IL-8. IL-8 is a powerful neutrophil attractant, and is one of the major mediators of the inflammatory response. It promotes neutrophil chemotaxis and degranulation leading to local inflammation in damaged tissues^37^. Enhanced IL-8 expression production of both normal human keratinocytes and HaCaT cells was observed after stimulation *in vitro* by both irritants, sensitizer and tolerogen. This suggests that IL-8 may play a critical role in the early response to immunogenic and inflammatory signals with its response to nonspecific stimuli^38^. It was suggested that the differentiated production of IL-1α and IL-8 may be linked to the type of product applied, either an irritant or a sensitizer^39^,^40^. Our study showed that CuCl_2_ and Cu(OAc)_2_ had significantly upregulated HaCaT cell expression of IL-1α and IL-8 at both the gene level and protein level. The highest fold change in the gene level - approximately 44 times higher in IL-8 expression, was observed in 580 μM CuCl_2_ treated cells when compared to control. The upregulation of these cytokines will activate and promote an inflammatory cascade, leading to various signs and symptoms.

Besides the interleukin family, other genes related to the skin inflammatory mechanism were also upregulated. FOSL1 is an early gene that belongs to the activator protein 1 family of dimeric transcription factor genes^41^. It plays a role in the angiogenesis and vasculogenesis processes, where it regulates the expression of key molecules on the cell surface to modulate cell adhesion and motility^42^. While, HSPA1A belongs to the HSP70 family of heat shock proteins. It can be highly activated by various stress stimuli, where it is required for the re-folding of damaged and unfolded proteins generated under stress^43^. Both FOSL1 and HSPA1A are stress-inducible and our results showed that they were significantly upregulated in the presence of CuCl_2_ and Cu(OAc)_2_. Hence, FOSL1 and HSPA1A can potentially serve as useful inflammatory markers for copper compounds.

We have also studied other genes, SOD1, CFL1, BMP2 and HSP27 that were previously shown to be upregulated in the presence of sodium lauryl sulfate by DNA array or proteomic analysis^24^,^25^,^28^. However, our results showed that no significant upregulation were observed after treatment with various copper compounds (SI1). In previous studies, human keratinocytes, human epidermis model and human reconstructed skin model human reconstructed skin model were used for testing the response of sodium lauryl sulfate. These new endpoints have not been widely validated for skin irritation responses. Our study may prove that these endpoints are not suitable for irritancy testing of copper compounds in a monolayer HaCaT assay.

Copper delivery through skin can potentially be useful. However, the choice of copper compound remains important. At the same concentration, different copper compounds can exhibit different irritancy potential. From our results, we observed that GHK-Cu exhibits low potential of inducing skin irritation response in comparison with CuCl_2_ and Cu(OAc)_2_. GHK-Cu can potentially be useful in providing a safe delivery of copper through the skin to exert its beneficial effects. Copper salts are discouraged for direct use and application on the skin due to its irritation potential and toxicity. There are several mechanisms proposed in attempt to explain copper irritancy. Copper may become potential irritants when they are oxidized and causes the formation of free acids. It is the oxidizing action of such acids that potentially result in skin irritation reactions once they reach the viable skin layers^3^. It can also interact with reduced oxygen species to form hydroxyl radical that inactivates enzymes and disrupts the membranes and organelles^44^.

Interestingly, GHK-Cu did not show any signs of irritation. This may be due to the complexation of copper to the small biologically active peptide, GHK^45^. When GHK is coupled with copper, the peptide may quench the redox activity of copper, facilitating the non-toxic delivery of copper into the cells^46^. Complexation using other ligands can also be explored but GHK is commonly used in cosmetics with properties such as the stimulation of collagen synthesis, chemotaxis, anti-stinging effects and others^47^. Our results also showed that GHK itself is not toxic and does not induce the change of irritancy/sensitizing related biomarkers, which provides an additional benefit when used for complexation with copper.

Accurate evaluation of cosmetological, dermatological and pharmaceutical products after application on human skin are essential and crucial in product development. *In vitro* skin irritation test provides an alternative to animal testing and allows the screening of compounds before they undergo clinical testing. The current standard operating procedure by the European Center for the Validation of Alternative Methods (ECVAM) recommends the use of MTT reduction as parameter to make prediction on the skin irritancy of substances using EpiSkin^TM^ test method, while IL-1α endpoint is regarded as a useful adjunct to MTT assay^48^. However, measuring cytotoxicity alone is not sufficient. We have shown that CuCl_2_ and Cu(OAc)_2_ irritation induces the upregulation of various genes without the concomitant inhibition of cell viability. To provide a more accurate prediction of skin irritancy potential of the test compound, *in vitro* skin irritation test can include other potential biomarkers.

In this study, we evaluated various copper compounds at cellular, genomic and proteomic levels using HaCaT keratinocytes. For the first time, we have shown that different types of copper compounds have different irritancy potential at three different levels. Specifically, GHK-Cu was found to be least possible to cause skin irritation, while CuCl_2_ and Cu(OAc)_2_ were found to induce the expression of various skin irritation biomarkers, i.e., IL-1α, IL-8, FOSL1, and HSPA1A. These biomarkers can be used in adjunct to MTT assay for a more accurate prediction of the *in vivo* response. This irritancy test approach may be extended to other similar compounds or minerals and can potentially predict the *in vivo* skin irritation response before conducting clinical testing on human subjects.

Further investigations can include a validation study of the genes involved in the skin irritation mechanism following direct treatment to cells. It is also necessary to confirm the safety of GHK-Cu on human subjects. This will allow us to determine the correlation of the *in vitro* results with the actual *in vivo* responses.

## Materials and Methods

### Materials

GHK and GHK-Cu were purchased from McBiotec (Nanjing, China). The ratio of GHK to Cu is 2:1 according to manufacturer data. Copper (II) chloride, 99%, and copper (II) acetate monohydrate, ACS reagent, ≥98%, powder were purchased from Sigma-Aldrich (St. Louis, USA). Phosphate buffered saline (PBS) (pH 7.4, 10×) was obtained from Vivantis (Malaysia). Dulbecco’s modified Eagle’s medium (DMEM), fetal bovine serum, random primers and SYBR safe DNA gel stain were supplied by Invitrogen, Life Technologies (USA). Trypsin and penicillin/streptomycin solution were obtained from PAN-Biotech GmbH (Germany). 3-(4,5-dimethylthiazol-2-yl)-2,5-diphenyl tetrazolium bromide (MTT) and dimethyl sulfoxide (DMSO) were acquired from MP Biomedicals (Illkirch, France). RNeasy Mini Kit and QuantiFast SYBR Green PCR kit were purchased from Qiagen (Germany). Random primers and avian myeloblastosis virus reverse transcriptase were purchased from Promega (Madison, Wisconsin, USA). Human interleukin 1 alpha and interleukin 8 enzyme-linked immunosorbent assay (ELISA) kits were purchased from Biolegend (San Diego, CA, USA). Human Fos-related antigen 1 (FOSL1) and Heat Shock 70 kDa Protein 1A (HSPA1A) ELISA kits were purchased prom MyBioSource (San Diego, CA, USA). All chemicals were used as supplied.

### pH Determination in Water and Culture Medium

GHK, GHK-Cu, Cu(OAc)_2_ and CuCl_2_ samples were prepared and diluted in sterile water and culture medium (DMEM supplemented with 10% fetal bovine serum and 1% penicillin/streptomycin) respectively to a final concentration of 580 μM and 5800 μM. The pH value was then recorded using the S220 SevenCompact^TM^ pH/Ion meter (Mettler Toledo, Switzerland).

### Cell Viability Assay

The cell viabilities of different concentrations of GHK, Cu(OAc)_2_ and CuCl_2_ against Human adult low Calcium high Temperature (HaCaT) keratinocytes were studied by MTT assay in 6 replicates. Cells were seeded at a density of 5000 cells per well in 200 μl fresh culture medium (DMEM supplemented with 10% fetal bovine serum and 1% penicillin/streptomycin solution) into 96-well flat-bottomed microtitre plates (Costar, Corning, USA) and incubated at 37 °C in humidified 5% CO_2_ using AutoFlow NU-5510 Direct Heat CO_2_ Incubator (NuAire, USA) for 24 hours before treatment. The culture medium was then removed after 24 hours incubation. Subsequently, 180 μl fresh culture medium and 20 μl of samples (0.058–58000 μM in sterile water) were added per well and incubated for 24, 48 and 72 hours. For control group, 180 μl fresh culture medium and 20 μl of sterile water were added. At the respective analysis point, the medium was removed, washed with 200 μl PBS and replenished with 200 μl fresh medium per well. Twenty μl of filtered MTT solution (5 mg/ml in PBS) was added to each well, after which the plates were incubated for an additional 4 h. The supernatant was then removed and resultant formazan crystals were solubilized in 150 μl DMSO. Absorbance was recorded at 595 nm with a microplate reader (Tecan, Switzerland). Wells containing DMSO alone were used as the blank. Percentage of cell viability was expressed as (A_sample_ − A_DMSO_)/(A_control_ − A_DMSO_) ×100%.

### Lactate Dehydrogenase (LDH) Assay

The relative amounts of LDH released into the media (conducted in triplicates for each group) were determined using CytoTox-ONE Homogeneous Membrane Integrity Assay kit Cat.#G7892 (Promega, USA) according to the manufacturer’s instructions. Briefly, HaCaT keratinocytes were seeded at a density of 10,000 cells per well in 200 μl fresh culture medium (DMEM supplemented with 10% fetal bovine serum and 1% penicillin/streptomycin solution) into 96-well flat-bottomed microtitre plates (Costar, Corning, USA) and incubated at 37 °C in humidified 5% CO_2_ using AutoFlow NU-5510 Direct Heat CO_2_ Incubator (NuAire, USA) for 24 hours before treatment. A set of no-cell control group containing only the culture medium without cells were prepared to serve as negative control to determine background fluorescence that might be present. Subsequently, 180 μl fresh culture medium and 20 μl of samples (0.0058–5800 μM in sterile water) were added per well and incubated for 30 minutes. For the vehicle control group and the maximum LDH release control group, 180 μl fresh culture medium and 20 μl of sterile water were added. After 30 minutes, the media was replaced with fresh serum-free media and incubated for further 24 hours. For the maximum LDH release control group, 4 μl of lysis solution (Promega, USA) was added into each well and incubated for 30 mins to initiate cell lysis prior to the next step. 40 μl of supernatant was removed from each treated well and transferred into a 96-well clear bottom black microplate (Costar, Corning, USA). The same volume of Cytotox-ONE Reagent (Promega, USA) was added into each well and the microplate was shaken for 30 secs to mix. The microplate was then incubated at 22 °C for 10 minutes. 20 μl of Stop Solution (Promega, USA) was added into each well and the microplate was shaken for 10 secs. The relative fluorescence intensities were measured at the excitation wavelength (λ_Ex_) of 560 nm and emission wavelength (λ_Em_) of 590 nm. The average fluorescence values of the culture medium background were subtracted from all experimental wells (samples and controls). The percent (%) cytotoxicity was determined using the following formula for each given experimental treatment: Cytotoxicity (%) = 100 ×  [(Experimental – Culture Medium Background)/(Maximum LDH Release – Culture Medium Background)].

### *In Vitro* Skin Irritation Test

HaCaT keratinocytes were cultured in DMEM containing 10% fetal bovine serum and 1% penicillin/streptomycin and were maintained under 37 °C with humidified 5% CO_2_. When the cells reached approximately 80% confluency, cells were harvested with trypsin and seeded at a density of 2.9 × 10^5^ cells per well in 2.5 ml of culture medium into 6 well flat-bottomed plates (Costar, Corning, USA) and incubated for 24 hr.

GHK, CuCl_2_ and Cu(OAc)_2_ were used at a final concentration of 58 μM and 580 μM. Sterile water was used as a control. All the chemicals were diluted with sterile water to appropriate concentrations just before the start of each experiment.

Then, 250 μl sample solution (580 and 5800 μM in sterile water) and 2.25 ml of culture medium were added into each well in triplicates. For control group, 250 μl sterile water and 2.25 ml culture medium were added. Cells were subjected to treatment in incubator for 24 hr. Following treatment, the culture medium was recovered and used for protein quantification using ELISA. The cells in the appropriately labelled wells were used subsequently for messenger ribonucleic acid (mRNA) extraction.

### mRNA Extraction

mRNA were extracted from cells using the RNeasy Mini Kit in accordance with the manufacturer’s instructions. The concentration of mRNA was determined using NanoDrop 1000 Spectrophotometer (Thermo Scientific, USA). Reverse transcription of total mRNA were performed at 1 μg of total mRNA in 25 μl final volume using random primers and avian myeloblastosis virus reverse transcriptase. The concentration of complementary deoxyribonucleic acid (cDNA) after reverse transcription were also determined using NanoDrop Spectrophotometer.

### Quantitative Real-Time Polymerase Chain Reaction (PCR)

Quantitative real-time PCR reaction was performed using Rotor-Gene Q real time PCR cycler (Qiagen, Germany). Primers (Integrated DNA technologies, Singapore) used for PCR reactions were listed in Table 1. Primers sequence for beta actin (ACTB), IL-1α, FOSL1, CFL1, BMP2 and HSPA1A were synthesized as previously described^24^,^25^,^49^. While primers sequence for IL8, HSP27 and SOD1 were designed using Primer3 (http://frodo.wi.mit.edu/) and Primer-BLAST (http://www.ncbi.nlm.nih.gov/tools/primer-blast/). Primer specificity was verified by running PCR for 40 cycles at 95 °C for 10 seconds and 60 °C for 60 seconds, followed by gel electrophoresis on a 3% agarose gel stained with SYBR safe DNA gel stain.

A beta actin primer was also included as an internal loading control. Each reaction mixture was prepared using 10 μl QuantiFast SYBR Green PCR master mix, 4 μl of cDNA template with 1 μM of each primer in a total reaction volume of 20 μl. The PCR was run for 40 cycles and the thermal cycling conditions were as follows: initial heat activation at 95 °C for 10 minutes; denaturation for 10 seconds at 95 °C; combined primer annealing and extension for 60 seconds at 60 °C. The fluorescence signal was measured at the end of each extension step. After the amplification, a melting peak analysis with a temperature gradient from 72 °C to 95 °C was performed. Fluorescence emission readings were analyzed using Rotor-Gene Q software (Qiagen, Germany). The data were presented as the fold increase of the target gene expression, normalized to the housekeeping gene beta-actin, compared to unstimulated conditions.

### ELISA

Following treatment, conditioned medium was recovered and used to determine concentration of IL-1α, IL8, HSPA1A and FOSL1. The concentration of IL-1α and IL8 were measured quantitatively using ELISA kits in accordance with the manufacturer’s instructions. Absorbance was recorded at 450 nm using a microplate reader (Tecan, Switzerland). The unknown analyte concentrations in the samples were determined using a standard curve. The concentration range of standard curve for IL-1α is 3.9–250 pg/ml, for IL8 15.6–1000 pg/ml, for FOSL1 31.2 to 1000 pg/ml and for HSPA1A 1.56–100 ng/ml. Results were presented as pictogram or nanogram of mediator released per milliliter of conditioned medium.

### Statistical Analysis

Results were expressed as mean ± standard deviation of at least three independent experiments. Statistical analysis was performed by one-way analysis of variance (ANOVA) followed by Tukey’s *post hoc* test using Minitab 16 and Student’s t-test. The difference was considered to be statistically significant at *p*-value < 0.05.

## Additional Information

**How to cite this article**: Li, H. *et al*. Selected Biomarkers Revealed Potential Skin Toxicity Caused by Certain Copper Compounds. *Sci. Rep.* **6**, 37664; doi: 10.1038/srep37664 (2016).

**Publisher's note:** Springer Nature remains neutral with regard to jurisdictional claims in published maps and institutional affiliations.

## Supplementary Material

Supplementary Information

## Figures and Tables

**Table 1:** DNA sequence of primer pairs used for quantitative real time PCR.

Gene symbol	Gene name	Accession Number	Product length (bp)	Forward Primer (5′ to 3′)	Reverse Primer (5′ to 3′)
IL1A	Interleukin 1 alpha	NM_000575	89	ggttgagtttaagccaatcca	tgctgacctaggcttgatga
FOSL1	FOS-like antigen 1	NM_005438	75	aaccggaggaaggaactgac	ctgcagcccagatttctcat
HSPA1A	Heat shock 70 kDa protein 1A	NM_005345	89	ggagtcctacgccttcaaca	ccagcaccttcttcttgtcg
BMP2	Bone morphogentic protein 2	NM_001206	189	ggtggaatgactggattg	gcatcgagatagcactg
CFL1	Cofilin 1	NM_005507	285	tcttctgcctgagtgaggac	tgatccctgtcagcttcttc
IL8	Interleukin 8	NM_000584	139	tctggcaaccctagtctgcta	agtgcttccacatgtcctcac
HSP27	Heat shock protein 27	AB020027	153	ctgcaaaatccgatgagactg	caggtggttgctttgaacttt
SOD1	Superoxide dismutase 1	NM_000454	166	tcaatttcgagcagaaggaaa	ccaccgtgttttctggataga
ACTB	Actin, beta	NM_001101	283	tgacccagatcatgtttgag	ttaatgtcacgcacgatttcc
