# Supplementary material for: Selected Biomarkers Revealed Potential Skin Toxicity Caused by Certain Copper Compounds
Source: Sci Rep. 2016 Nov 28;6:37664. doi: 10.1038/srep37664 (PMC5124859; doi:10.1038/srep37664)
Supplement: Supplementary Information [file srep37664-s1.pdf]

Selected Biomarkers Revealed Potential Skin Toxicity Caused by Certain Copper Compounds

Hairui Li[1, 2, #], Pei Zhen Toh[1, #], Jia Yao Tan[1,#], Melvin T. Zin[2], Chi-Ying Lee[2], Bo Li[2], Melvina Leolukman[2], Hongqian Bao[2], Lifeng Kang*

[1]Hairui Li, Pei Zhen Toh, Jia Yao Tan and Lifeng Kang

Department of Pharmacy, National University of Singapore, 18 Science Drive 4, Singapore 117543

[2] Hairui Li, Melvin T. Zin, Chi-Ying Lee, Bo Li, Melvina Leolukman and Hongqian Bao

3M Innovation Singapore, 100 Woodlands Avenue, Singapore 738205

[#]Hairui Li, Pei Zhen Toh and Jia Yao Tan

These authors contributed equally to this work.

* Corresponding author: Dr. Lifeng Kang, Department of Pharmacy, National University of Singapore, 18 Science Drive 4, Singapore 117543. Tel: + 65 6516 7519, Fax: +65 6779 1554. E-mail address: lkang@nus.edu.sg

Supplementary Information

SI 1. The fold change in gene levels after treatment in HaCaT cells. HaCaT keratinocytes were treated with sterile water, 58 µM and 580 µM of GHK, GHK-Cu, $Cu(OAc)_2$ and $CuCl_2$ for 24 hours in triplicates. The changes in relative gene expression were calculated as the normalized ratio in treatment cells compared to that in control (sterile water). Fold change in treated cells are presented as mean ± standard deviation. ANOVA was performed between the control and treatment groups for each gene followed by Tukey's post hoc test. * $p < 0.05$, is considered to be statistically significant.

Gene symbol	GHK 58uM	GHKCu 58µM	CuCl2 58µM	CuOAc 58uM	GHK 580uM	GHKCu 580µM	CuCl2 580µM	CuOAc 580uM
IL1A*	1.21 ± 0.39	0.71 ± 0.14	1.07 ± 1.01	1.37 ± 0.37	1.24 ± 0.28	3.05 ± 1.33	8.57 ± 4.23	11.30 ± 2.16
IL8*	1.83 ± 0.89	1.73 ± 0.90	2.35 ± 1.19	1.26 ± 0.15	2.47 ± 0.98	2.97 ± 0.88	44.07 ± 8.74	11.22 ± 2.82
FOSL1*	1.12 ± 0.22	2.53 ± 1.85	3.53 ± 2.27	1.37 ± 0.14	0.95 ± 0.15	3.04 ± 1.56	17.40 ± 3.89	9.35 ± 1.58
HSPA1*	3.12 ± 1.56	0.46 ± 0.03	2.64 ± 2.09	1.26 ± 0.32	2.02 ± 1.29	0.64 ± 0.11	18.23 ± 2.60	8.27 ± 3.57
SOD1	0.37 ± 0.07	0.50 ± 0.07	1.00 ± 1.14	0.46 ± 0.24	0.37 ± 0.08	0.56 ± 0.09	0.85 ± 0.16	0.36 ± 0.14
HSP27	0.23 ± 0.02	0.41 ± 0.18	0.42 ± 0.21	0.81 ± 0.43	0.21 ± 0.06	0.55 ± 0.16	0.11 ± 0.04	0.53 ± 0.36
BMP2	0.34 ± 0.06	1.81 ± 1.29	1.52 ± 1.12	0.17 ± 0.03	0.25 ± 0.13	2.94 ± 2.97	1.03 ± 0.82	0.22 ± 0.05
CFL1	0.13 ± 0.06	2.57 ± 2.13	1.56 ± 1.45	0.11 ± 0.05	0.16 ± 0.06	4.50 ± 3.89	0.51 ± 0.37	0.06 ± 0.02

SI 2. pH of tested compounds dissolved in water and culture medium respectively

Concentration / Solvent	5800 μM Water	5800 μM Culture medium	580 μM Water	580 μM Culture medium
GHK	7.95	7.60	7.92	7.49
GHK-Cu	6.74	7.23	6.41	7.35
CuCl2	4.69	6.91	5.09	7.21
Cu(OAc)2	5.87	6.92	5.56	7.37
